# Fibrosing Mediastinitis: Uncommon Life-threatening Complication of Histoplasmosis

**DOI:** 10.7759/cureus.2532

**Published:** 2018-04-25

**Authors:** Muhammad Khalid, Imran Khan, Zia Rahman, Ahmad Alazzeh, Dima Youssef

**Affiliations:** 1 Department of Internal Medicine, East Tennessee State University, Quillen College of Medicine, Johnson City, USA

**Keywords:** histoplasmosis, lymphadenopathy, calcification

## Abstract

Histoplasmosis involving mediastinum is very rare which can present as a mediastinal mass or fibrosing mediastinitis. Fibrosing mediastinitis can be life-threatening if left untreated due to the involvement of the surrounding visceral and vascular structures. We present an interesting case of fibrosing mediastinitis due to histoplasmosis presented with palpitations, chest pain and dyspnea. The patient had mediastinal and hilar lymphadenopathy with calcification on chest imaging. The patient was diagnosed on lymph node biopsy and treated with antifungals.

## Introduction

Fibrosing mediastinitis is a rare disorder characterized by an excessive fibrotic reaction in the mediastinum. It usually results from an excessive host response to a prior infection that involves mediastinal lymph nodes. The vast majority of cases are thought to be sequelae of Histoplasma capsulatum infection. However, it is a relatively uncommon presentation of histoplasmosis resulting from leakage of fungal antigens from lymph nodes into the mediastinal space, leading to a hypersensitivity reaction followed by an exuberant fibrotic response. We present a rare case of 19-year-old healthy male who has fibrosing mediastinitis due to histoplasmosis.

## Case presentation

**Figure 1:**
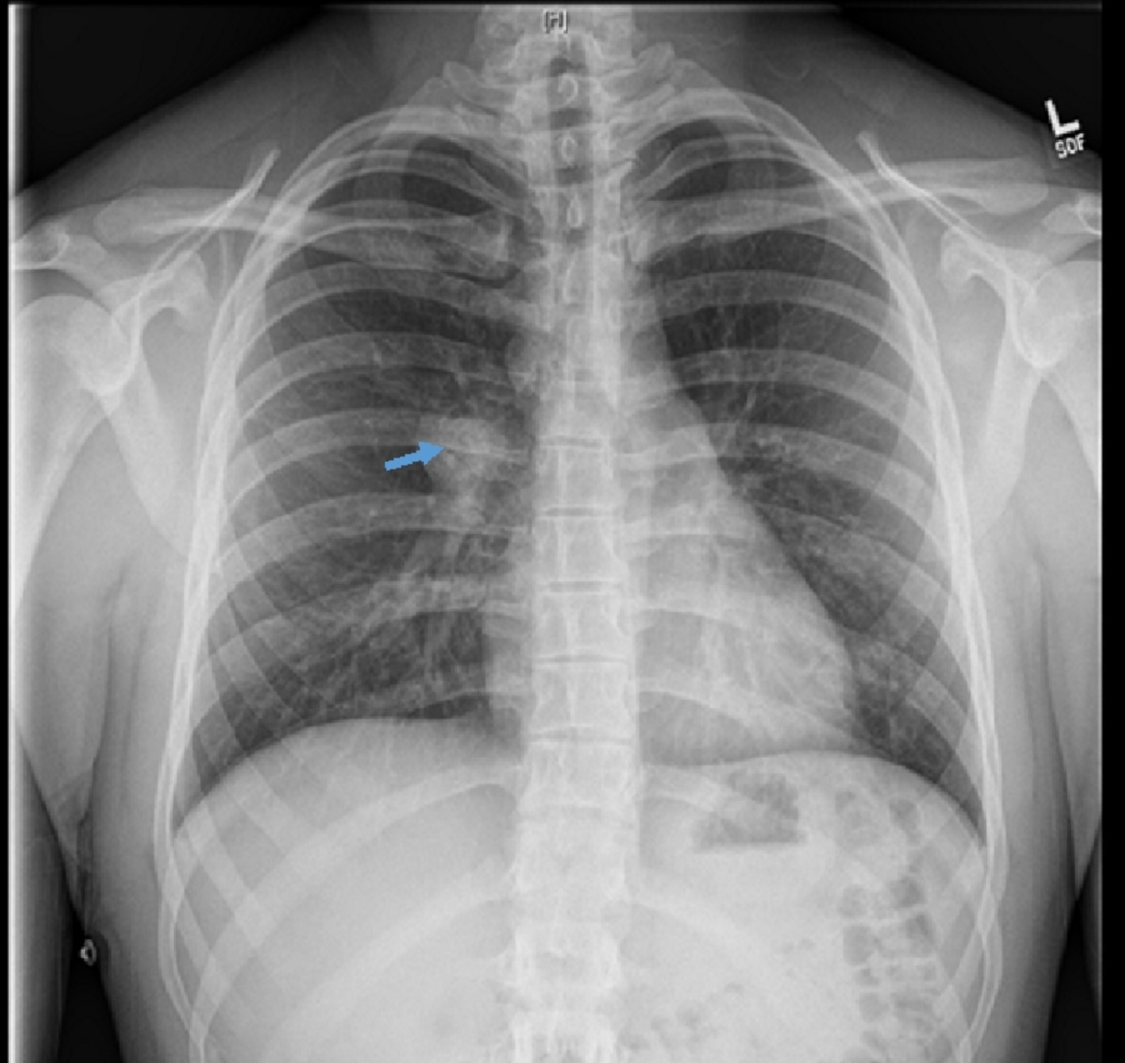
Chest X-ray showed perihilar mass (Arrow).

A 19-year-old healthy male with no significant medical history presented with complaints of palpitations for one day along with chest pain and shortness of breath. It started when he was working on a Christmas tree farm. The patient reported a history of similar episodes for last six years usually triggered with mild exertion. He had noticed increased frequency and severity of symptoms for last six months and it started happening at least twice per week. He denied smoking, drinking alcohol, excessive caffeine consumption, substance use, recent travel, cough, fever, chills, night sweats, hemoptysis, weight loss, joint pain, rash, nausea, vomiting, abdominal pain and syncopal episodes. The patient has been feeling more fatigued and tired for last few months. On admission, vitals were normal. On physical examination, he was anxious and appeared to be in distress, rest of the systemic examination was unremarkable. Laboratory results showed normal liver, kidney functions and troponins. Urine drug screen was negative. Chest X-ray showed a right perihilar mass of 3.1 x 3.4 cm (Figure 1).

**Figure 2:**
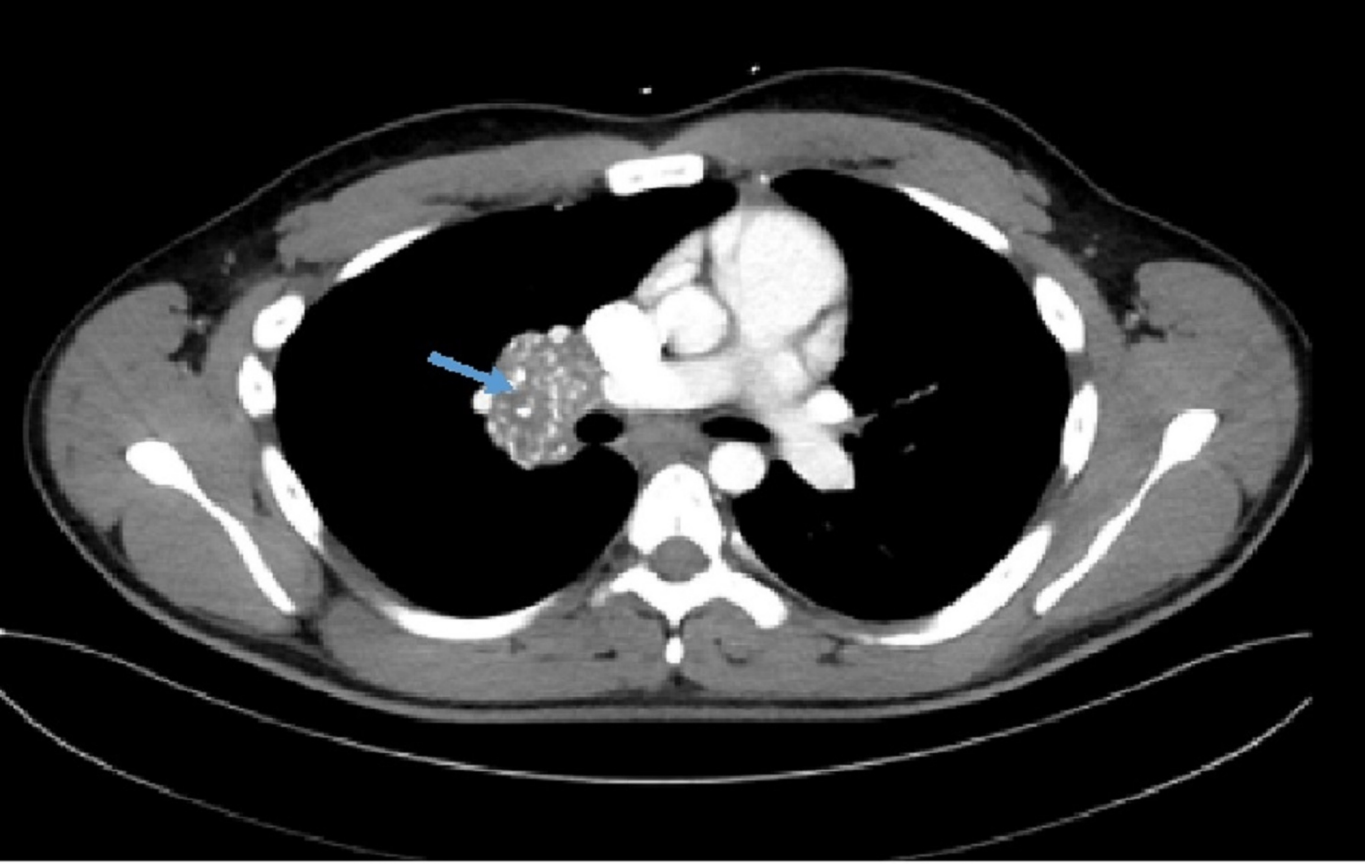
Computed tomography (CT) chest showed perihilar mass (Arrow).

Computed tomography scan of the chest showed stippled-type calcified mediastinal lymphadenopathy of 1.6 cm x 2.1 cm, and right hilar mass of 3.8 x 4.8 cm (Figure 2).

The patient had a bronchoscopy with fine needle aspiration (FNA) of mediastinal mass and lymph nodes. FNA results came back negative for malignancy, gram stain and cultures, acid-fast bacilli (AFB) stain and cultures, fungal stain and cultures were all negative. Histological examination showed predominant necrosis with rare benign lymphoid tissue. Urine histoplasma antigen and serum Aspergillus galactomannan antigens were negative. The patient continued to have chest discomfort and dyspnea after bronchoscopy. He had a mediastinoscopy with lymph node biopsy which showed an inflammatory granulomatous process. Pathology report showed lymphoid tissue with necrotizing granulomatous inflammation and positive fungal stain and morphology consistent with histoplasma species. Fungal and AFB cultures remained negative. The patient was started on oral itraconazole 200 mg twice a day with a plan for regular outpatient follow-up. He had improvement in chest symptoms during routine infectious disease clinic follow-up. He could not tolerate itraconazole due to diffuse rash after two weeks of treatment. He was switched to oral fluconazole 400 mg once daily which he tolerated without any problems.

## Discussion

Fibrosing mediastinitis, also known as sclerosing mediastinitis is a rare disorder characterized by dense fibrous tissue infiltration within mediastinum. It is an idiosyncratic reaction to Histoplasma capsulatum infection, immunoglobulin G4 (IgG4)-related disease or drug-related toxicity [1]. It is reported as abnormal host immunological response to histoplasma responsible for fibrosis. The study by Strock et al. reported an association of major histocompatibility complex, class II, DQ beta 1 *04:02 subtype with post histoplasmosis fibrosing mediastinitis [2]. Histoplasmosis involving mediastinum is very rare which can present as a mediastinal mass or fibrosing mediastinitis [3]. There are two different histopathological type of fibrosing mediastinitis, focal granulomatous and non-granulomatous form. Granulomatous form of fibrosing mediastinitis is due to immune-mediated hypersensitivity reaction which has been reported with histoplasma infection especially in North America [2, 4].

The clinical presentation is diverse and depends upon the involvement of surrounding structures of the mediastinum. It can mimic malignancy and cause different complications due to compression of pulmonary and vascular structures, including superior vena cava syndrome [2] and esophagus. Cough, chest pain, dyspnea, hemoptysis or pneumonia is the most common presentation. Dunn et al. reported two cases of fibrosing mediastinitis with massive hemoptysis requiring pneumonectomy from bronchial artery due to erosion [5].

There is significant overlap between fibrosing mediastinitis and IgG4-related disease. The study done by Peikert et al. reported one-third of patients with granulomatous fibrosing mediastinitis or histoplasmosis showed histopathological features of IgG4-related disease [6]. Fibrosing mediastinitis is diagnosed incidentally in 40% of cases.

Diagnosis needs imaging studies to confirm the presence of an infiltrative process in the mediastinum, to help exclude malignancy, and to assess the integrity of mediastinal structures and biopsy is required to confirm the diagnosis. Chest radiography usually shows calcified mediastinal mass like in our case patient who had a right perihilar mass. Bronchoscopy and lymph node biopsy can be helpful to confirm the diagnosis. Most of the patients have known histoplasmosis, but our patient never had known infection and was not taking any immunosuppressant.

There are no specific treatment guidelines available for fibrosing mediastinitis. Stents placement by bronchoscopy or percutaneously placed vascular stents have been used for ameliorating central airway and vascular obstruction, respectively. Surgery can be performed to palliate symptoms by relieving airway, vascular, and esophageal obstruction. Cases have been reported about hemoptysis due to histoplasmosis-related mediastinal fibrosis resulted in superior vena cava syndrome, treated by bronchial artery embolization and transcatheter percutaneous stent placement [7, 8]. Prognosis is good in most of the cases but it could be life-threatening due to massive hemoptysis, recurrent postobstructive pneumonia due to bronchial compression, pulmonary and heart disease [9].

Our patient had positive lymph node biopsy for histoplasmosis even after initial negative FNA results. This case highlights the importance of early diagnosis of mediastinal involvement of histoplasmosis and appropriate treatment with antifungal agent can prevent fatal and dreadful consequences.

## Conclusions

Fibrosing mediastinitis is a rare complication of histoplasmosis in young patients which can present in different ways due to the compression of surrounding visceral and vascular structures. It can be life-threatening if left untreated. Travel and exposure history can be helpful to narrow the differential diagnosis. Timely diagnosis and intervention can prevent fatal consequences.

## References

[1] Patel M, Lu F, Hannaway M, Hochman K (2015). Fibrosing mediastinitis: a rare complication of histoplasmosis. BMJ Case Rep.

[2] Strock SB, Gaudieri S, Mallal S (2015). Fibrosing mediastinitis complicating prior histoplasmosis is associated with human leukocyte antigen DQB1*04:02 - a case control study. BMC Infect Dis.

[3] Shersher DD, Hong E, Breard J, Warren WH, Liptay MJ (2012). Anterior mediastinal mass secondary to histoplasmosis. Ann Thorac Surg.

[4] McKinsey DS, McKinsey JP (2011). Pulmonary histoplasmosis. Semin Respir Crit Care Med.

[5] Hage CA, Wheat LJ, Loyd J, Allen SD, Blue D, Knox KS (2008). Pulmonary histoplasmosis. Semin Respir Crit Care Med.

[6] Peikert T, Shrestha B, Aubry MC (2012). Histopathologic overlap between fibrosing mediastinitis and IgG4-related disease. Int J Rheumatol.

[7] Johansen M, Hoyer M, Kleiman M (2013). Transcatheter treatment of SVC syndrome from histoplasmosis-related mediastinal fibrosis in a 9-year old male. Catheter Cardiovasc Interv.

[8] Thomas BP, Bream PR Jr, Milstone AP, Meranze SG (2006). Treatment of SVC syndrome and hemoptysis in a patient with mediastinal fibrosis. Emerg Radiol.

[9] Peikert T, Colby TV, Midthun DE, Pairolero PC, Edell ES, Schroeder DR, Specks U (2011). Fibrosing mediastinitis: clinical presentation, therapeutic outcomes, and adaptive immune response. Medicine.

